# Supplementary material for: Can SMEs synergistic transformation of digitization and greenization promote enterprise risk-taking?
Source: PLoS One. 2025 Dec 11;20(12):e0337815. doi: 10.1371/journal.pone.0337815 (PMC12698014; doi:10.1371/journal.pone.0337815)
Supplement: Attachment — Submitted filename: Response to Reviewers.docx [file pone.0337815.s001.docx]

Dear academic editor and reviewers:

Thank you for taking the precious time to review my manuscript. These valuable comments are very helpful for improving the quality of the paper. Over the past six weeks, I have revised the abstract, contributions, model and other contents of the paper in accordance with these commets. These revisions are of great help to the quality of the paper. The responses to the editor and reviewers are as follows:

1. **To academic editor**

**Additional Editor Comments : In addition to the reviewers suggestions, I encourage you to include in your manuscript how you deal with potential endogeneity problems in your data and models.**

**The response of author：**
Thank you for the comments of the academic editor. Although the fixed effects model is used in this paper, there are still possible endogeneity problems. We have incorporated the academic editor's suggestions for additional endogeneity test. In order to determine the possible endogeneity problems, this paper uses the degree of synergistic transformation of digitization and greenization in the city where the enterprise is located (CITYSDG) as an instrumental variable, and uses the hausman test to verify whether the model has endogeneity. A section "Endogeneity test" （line 728-741）has been added to the "Empirical results and analysis" of the paper. It is expressed as follows:

**Endogeneity test**

In this paper, the Hausman test determines whether the model has endogeneity problems. This paper draws on the study of Luo ^[71]^. It uses the degree of synergistic transformation of digitization and greenization in the city where the enterprise is located (CITYSDG) as an instrumental variable. According to the first law of geography, everything is related to everything else. However, close things are more related than far away ^[72]^. Therefore, the degree of CITYSDG affects SDG but does not directly affect enterprise risk-taking. Moreover, the data at the regional level are complex. They can be affected by the characteristics of enterprises and meet the exclusion constraints. Moreover, the underidentification test (P value=0.0000) and weak identification test (F=23.01>10%10% critical value) are conducted in this paper, and the results show that CITYSDG is a qualified instrumental variable. Hausman test results show that Prob>chi2 = 1.0000, indicating that the fixed effect model used in this paper does not have significant endogeneity problems.

2. **To reviewer 1：**

**(1) The research model is simple and lacks in-depth discussion for their research. The abstract is overly focused on the background information, which fails to adequately highlight the novelty and significance of their research. The abstract should be improved to emphasize the study’s contributions and its broader implications.**

**The response of author：**

Thank you to the reviewers for your suggestions on the abstract writing. Highlighting the contribution and significance of the research in the abstract is of great importance for readers to understand the main content of the paper. To highlight both of them, the author summarized the contribution and significance of the paper and briefly described them in the abstract. The main changes are as follows (line 12-33):

The synergistic transformation of digitization and greenization has become an important direction in China’s economic development. Small and Medium Enterprises (SMEs) have the characteristics of small scale and high growth and have become the key force of synergistic transformation of digitization and greenization. Synergistic transformation of digitization and greenization helps to promote the coordination and unification of SMEs’ operating efficiency and environmental protection, resulting in the effect of 1+1>2, which has become an important factor in promoting enterprise risk-taking. Using the sample of enterprises listed on the SME board from 2012 to 2022, this paper adopts the Haken model to measure SMEs’ synergistic transformation of digitization and greenization and analyzes its impact on enterprise risk-taking, drawing the following conclusions: (1) SMEs’ synergistic transformation of digitization and greenization significantly promotes enterprise risk-taking. (2) The impact mechanism analysis finds that SMEs’ synergistic transformation of digitization and greenization improves enterprise risk-taking by reducing cash holdings and Research and Development (R&D) investment. (3) Further research finds that compared with state-owned SMEs, a synergistic transformation of digitization and greenization of non-state-owned SMEs has a more significant impact on enterprise risk-taking. Compared with high-tech SMEs, the synergistic transformation of digitization and greenization of non-high-tech SMEs has a more significant impact on enterprise risk-taking. These conclusions have practical significance for promoting SMEs’ synergistic transformation of digitization and greenization and improving enterprise risk-taking.

(2) **Literature Review and introduction lacks enough latest references to help the readers understand their research, the current version is difficult to raise concerns about the article’s originality and relevance.**

**The response of author：**

We highly agree with the reviewers' comments on the literature review. This comment is very helpful for improving the writing of the literature review in this papers. Scholars' research on the impact of synergistic transformation of digitization and greenization on enterprise risk-taking is still relatively rare. In the existing studies, a considerable number of scholars have noticed that the enterprise digital transformation affects risk taking, while the impact of enterprise green transformation on risk taking has not attracted widespread attention from scholars. In the research on digitalization and greenization, most scholars pay more attention to the impact of digital transformation on green transformation. This paper precisely discovers this research gap, measures the degree of SMEs synergistic transformation of digitization and greenization using the Haken model, and analyzes its impact on the risk taking. In order to enable readers to better understand the research progress of this topic, the following revision have been made to this article:

①In order to better present the research progress in the relevant research field of this paper, this paper has retrieved the latest literature in the relevant field and updated the latest literature in the literature review. The main changes are as follows (line 174-195):

Scholars’ research on the synergistic transformation of digitization and greenization focuses on the impact of digital transformation on green transformation. Zhang ^[1]^ discovered that digital transformation promotes enterprise green transformation by alleviating information asymmetry. More scholars focus on the relationship between enterprise digital transformation and green technology innovation. For example, enterprise digital transformation can help alleviate financing constraints ^[2,27,28]^, attract government subsidies ^[3,5]^, increase R&D investment ^[5]^, and improve management efficiency. Lin & Xie ^[30]^ further enhance enterprise green technology innovation. Du ^[3]^ proposed that enterprise digital transformation alters enterprise factor allocation and energy production mode, promotes information transparency, reduces transaction costs, and helps promote energy technology innovation. On the other hand, Dou & Gao ^[4]^ pointed out that enterprise digital transformation helps promote green technology innovation. This impact is more significant for state-owned enterprises, manufacturing enterprises, and enterprises in regions with strict environmental supervision. Some scholars have also determined that greenization has a pulling effect on promoting the application of digital technology. For example, Yu ^[31]^ assessed that energy enterprises actively use digital technologies to improve their production processes and management systems to reduce pollutant emissions and avoid environmental violations. From the above analysis, it can be seen that digitalization and greenization have a mutually reinforcing effect. However, no scholars have measured the degree of synergistic transformation of digitization and greenization and analyzed its economic consequences. There is still a gap in this area of research.

②Based on the review of the original literature, the limitations of the existing literature and the theoretical contributions of this paper are added to enable readers to clearly understand the originality and relevance of this paper compared with the previous literature. The main expression is as follows (line 196-216):

Academics highly value the study of enterprise risk-taking, green transformation, and digital transformation since it helps us comprehend the financial effects of digitization and greenization. Firstly, enterprise digital transformation helps improve the efficiency of information transmission, organizational efficiency, and innovation ability, and it promotes enterprise risk-taking. Secondly, enterprise green transformation helps reduce agency costs, alleviate information asymmetry, and promote enterprise risk-taking. Thirdly, enterprise digital transformation helps promote green technology innovation and green transformation. Green transformation also has a pulling effect on the application of digital technology. However, the following problems remain unsolved in the existing research: Firstly, the existing literature does not pay attention to the measurement of synergistic transformation of digitization and greenization. This paper uses the Haken model to measure the SMEs’ synergistic transformation of digitization and greenization, further deepening the research in this field. Secondly, the existing literature analyzes the economic consequences of digital or green transformation from a single perspective. This study further explores the impact of SMEs’ synergistic transformation of digitization and greenization on risk-taking. Thirdly, the mediating effect of cash holdings and R&D investment in the impact of SMEs’ synergistic transformation of digitization and greenization on risk-taking is not apparent. This paper attempts to answer the above questions, extend the application scope of the Haken model, and provide empirical evidence on the impact of SMEs’ synergistic transformation of digitization and greenization on risk-taking.

(3) **The knowledge gap of their research in Literature Review is not clear for reader, indicating that it is difficult to understand their contributions or to provide a forward-looking perspective. The authors should revise this part.**

**The response of author：**

Based on the review of the original literature, the limitations of the existing literature and the theoretical contributions of this paper are added to enable readers to clearly understand the originality and relevance of this paper compared with the previous literature. The main expression is as follows (line 196-216):

Academics highly value the study of enterprise risk-taking, green transformation, and digital transformation since it helps us comprehend the financial effects of digitization and greenization. Firstly, enterprise digital transformation helps improve the efficiency of information transmission, organizational efficiency, and innovation ability, and it promotes enterprise risk-taking. Secondly, enterprise green transformation helps reduce agency costs, alleviate information asymmetry, and promote enterprise risk-taking. Thirdly, enterprise digital transformation helps promote green technology innovation and green transformation. Green transformation also has a pulling effect on the application of digital technology. However, the following problems remain unsolved in the existing research: Firstly, the existing literature does not pay attention to the measurement of synergistic transformation of digitization and greenization. This paper uses the Haken model to measure the SMEs’ synergistic transformation of digitization and greenization, further deepening the research in this field. Secondly, the existing literature analyzes the economic consequences of digital or green transformation from a single perspective. This study further explores the impact of SMEs’ synergistic transformation of digitization and greenization on risk-taking. Thirdly, the mediating effect of cash holdings and R&D investment in the impact of SMEs’ synergistic transformation of digitization and greenization on risk-taking is not apparent. This paper attempts to answer the above questions, extend the application scope of the Haken model, and provide empirical evidence on the impact of SMEs’ synergistic transformation of digitization and greenization on risk-taking.

**(4) The flowchart of the model is low quality which lacks a detailed description of the research process. The authors can add a paragraph to describe the main step of the method which may enhance readability of their model.**

**The response of author：**

Thank you to the reviewers for your comments on the model flowchart. Based on these comments, this article has revised Figure 1 to make it more detailed and provide readers with more information. Meanwhile, a paragraph has been added to help readers understand the main research framework of this article. The main modifications of this part are as follows (line 342-355):

Fig1. The Impact of SMEs’ Synergistic Transformation of Digitization and Greenization on Enterprise Risk-Taking

Fig. 1 illustrates the mechanism of the SMEs’ synergistic transformation of digitization and greenization on risk-taking. First of all, the system of digitization and greenization in SMEs promotes integrating technology and management, helping SMEs establish unique competitive advantages. These are powerful factors that promote enterprise risk-taking. Secondly, SMEs’ synergistic transformation of digitization and greenization improves the synchronous operation among internal organizations, reduces the cash holding by agency motivation and prevention motivation, and helps promote enterprise investment expenditure and risk-taking. Thirdly, the system of digitization and greenization in SMEs helps to improve the sharing of knowledge, reduce the investment scale of innovation investment per unit of output, improve the efficiency of R&D investment, and thus increase risk-taking.

(4) **Their policy implications do not provide specific recommendations based on the conclusions found in the paper's research, but rather broad policy implications. It means that the policy implications should have a close relationship with their results.**

**The response of author：**

Thank you for your comments on the suggestions. This paper has revised the suggestions to make it more in line with the characteristics of SMEs synergistic transformation of digitization and greenization. The main expressions are as follows (870-900):

Strong growth and innovation make SMEs important for China’s economic development. Under the background of the development of the digital economy and green economy, in order to promote the SMEs’ synergistic transformation of digitization and greenization and improve the level of enterprise risk-taking, this paper puts forward the following suggestions:

Firstly, local governments should formulate policies according to encourage SMEs synergistic transformation of digitization and greenization. Risk-taking is an important driving force for enterprises to achieve sustainable development. Local governments should formulate relevant policies in line with the characteristics of SMEs according to the development dynamics of their digital economy and green economy, such as financial subsidies, technical assistance, and other policies. It aims to help SMEs carry out the synergistic transformation of digitization and greenization, help enterprises transform and upgrade, and increase enterprise risk-taking.

Secondly, SMEs should improve the efficiency of capital utilization and reduce cash holdings. SMEs should use the synergistic system of digitization and greenization to establish a diversified information acquisition mechanism and build a digital platform to transmit green and sustainable development information to enterprises. It can establish a good corporate image for enterprises, attract more external funds to deepen the synergistic transformation of digitization and greenization, alleviate the agency problem between organizations, reduce the level of cash holdings, and improve the efficiency of capital utilization to promote the level of enterprise risk-taking.

Thirdly, SMEs should improve their technological level and the efficiency of R&D investment. SMEs should play a full role in the synergy between the digital and green subsystems and promote the efficiency of R&D investment through the deep integration of digital technology and green innovation. Using big data, Artificial Intelligence (AI), the Internet of Things, and other digital technologies to optimize the R&D process, SMEs could reduce trial and error costs. It improves the efficiency of R&D investment, accelerates the breakthrough of green technology, and finally achieves a synergistic development in production efficiency, environmental performance, and risk-taking, as well as builds core competitiveness in the wave of sustainable development.
**(6)The manuscript is recommended to be checked for English by a native speaker.**

**The response of author：**
Thank you to the reviewers for the comments on the language of the paper. In order to make the language more fluent, the author, in collaboration with an English professional translator, further revised the word choice, sentence structure and paragraphs of the entire text.

3. **To reviewer 2：**

**(1)Provide a clearer rationale for using the Haken model, including examples of its application in similar contexts.**

**The response of author：**

We thank the reviewer for the comments about Haken model, which has prompted us to re-evaluate and strengthen our arguments. Providing the principles of Haken model used plays a very important role in helping readers understand the results of this paper. The Haken model is a widely used model in synergitics. In order to enable readers to have a clearer understanding of the haken model, this paper adds an introduction to the Haken model in 4.3.3.1. Firstly, this paper explains the possibility of the synergitics in explaining the degree of SMEs synergistic transformation of digitization and greenization. Secondly, the Haken model was introduced. Thirdly, the application scenarios of using the Haken model for economic research were presented. The main expression is as follows (line 409-442):

Synergetics is the study of the process from imbalance to equilibrium in the development of affairs and has broad application prospects in social science ^[34]^. After the birth of synergetics, many scholars in social science research use this theory to explain the synergistic relationship between subsystems in an enterprise. For example, Guasmin & Rajindra ^[60]^ analyzed the synergistic action between human resource management, strategic marketing management, and financial performance. According to Synergetics, the synergistic transformation of digitization and greenization is an open, non-equilibrium system. The system has evolved from disorder to order since its emergence. Therefore, it is necessary to establish an order parameter equation to identify the degree of synergistic transformation of digitization and greenization of each enterprise. The Haken model describes how the subsystems of complex systems spontaneously form macroscopic ordered structures through nonlinear interactions and synergies. Without external intervention, the system can automatically evolve into an orderly structure or function according to specific laws. There are fast variables and slow variables in the system. Slow variables dominate fast variables, and the system’s long-term behavior depends on a few order parameters. Thus, slow variables should be found to determine the synergistic effect of two subsystems, and order parameter equations should be established. Nonlinear dynamics equations usually describe the Haken model. Its general form is

 (6)

where q is a variable that describes the system’s state; N is a nonlinear function reflecting the interaction between subsystems; α is the control parameter that determines the system’s stability; F(t) is a random fluctuating force. The Haken model is widely used in social science research. For example, Yang ^[13]^ selected five big cities in China as samples to analyze the dynamic co-evolution law of economy and logistics. It was found that metropolitan economic growth is the order parameter that promotes the coordinated development of the metropolitan economy and logistics. Zhong ^[14]^ analyzed and predicted the synergistic between economic growth and energy consumption in the Beijing-Tianjin-Hebei region. Du & Yang ^[61]^ analyzed the co-evolution law of the logistics industry and manufacturing industry in the Yangtze River Economic Belt, and discovered that the logistics industry plays an increasingly important role in the relationship between them. Based on the above research, this paper uses the Haken model to measure the degree of synergistic transformation of digitization and greenization. The calculation process is described as follows:

**(2) While the study acknowledges prior research on digital and green transformations individually, it could benefit from a more extensive discussion of studies that have examined their synergistic effects. Discuss prior research on the combined impact of digitization and greenization more extensively.**

**The response of author:**

We highly agree with the reviewers' comments on the literature review. This comment is very helpful for improving the writing of the literature review in this papers. Scholars' research on the impact of synergistic transformation of digitization and greenization on enterprise risk-taking is still relatively rare. In the existing studies, a considerable number of scholars have noticed that the enterprise digital transformation affects risk taking, while the impact of enterprise green transformation on risk taking has not attracted widespread attention from scholars. In the research on digitalization and greenization, most scholars pay more attention to the impact of digital transformation on green transformation. This paper precisely discovers this research gap, measures the degree of SMEs synergistic transformation of digitization and greenization using the Haken model, and analyzes its impact on the risk taking. In order to enable readers to better understand the research progress of this topic, the following revision have been made to this article:

①In order to better present the research progress in the relevant research field of this paper, this paper has retrieved the latest literature in the relevant field and updated the latest literature in the literature review. The main changes are as follows (line 174-195):

Scholars’ research on the synergistic transformation of digitization and greenization focuses on the impact of digital transformation on green transformation. Zhang ^[1]^ discovered that digital transformation promotes enterprise green transformation by alleviating information asymmetry. More scholars focus on the relationship between enterprise digital transformation and green technology innovation. For example, enterprise digital transformation can help alleviate financing constraints ^[2,27,28]^, attract government subsidies ^[3,5]^, increase R&D investment ^[5]^, and improve management efficiency. Lin & Xie ^[30]^ further enhance enterprise green technology innovation. Du ^[3]^ proposed that enterprise digital transformation alters enterprise factor allocation and energy production mode, promotes information transparency, reduces transaction costs, and helps promote energy technology innovation. On the other hand, Dou & Gao ^[4]^ pointed out that enterprise digital transformation helps promote green technology innovation. This impact is more significant for state-owned enterprises, manufacturing enterprises, and enterprises in regions with strict environmental supervision. Some scholars have also determined that greenization has a pulling effect on promoting the application of digital technology. For example, Yu ^[31]^ assessed that energy enterprises actively use digital technologies to improve their production processes and management systems to reduce pollutant emissions and avoid environmental violations. From the above analysis, it can be seen that digitalization and greenization have a mutually reinforcing effect. However, no scholars have measured the degree of synergistic transformation of digitization and greenization and analyzed its economic consequences. There is still a gap in this area of research.

②Based on the review of the original literature, the limitations of the existing literature and the theoretical contributions of this paper are added to enable readers to clearly understand the originality and relevance of this paper compared with the previous literature. The main expression is as follows (196-216):

Academics highly value the study of enterprise risk-taking, green transformation, and digital transformation since it helps us comprehend the financial effects of digitization and greenization. Firstly, enterprise digital transformation helps improve the efficiency of information transmission, organizational efficiency, and innovation ability, and it promotes enterprise risk-taking. Secondly, enterprise green transformation helps reduce agency costs, alleviate information asymmetry, and promote enterprise risk-taking. Thirdly, enterprise digital transformation helps promote green technology innovation and green transformation. Green transformation also has a pulling effect on the application of digital technology. However, the following problems remain unsolved in the existing research: Firstly, the existing literature does not pay attention to the measurement of synergistic transformation of digitization and greenization. This paper uses the Haken model to measure the SMEs’ synergistic transformation of digitization and greenization, further deepening the research in this field. Secondly, the existing literature analyzes the economic consequences of digital or green transformation from a single perspective. This study further explores the impact of SMEs’ synergistic transformation of digitization and greenization on risk-taking. Thirdly, the mediating effect of cash holdings and R&D investment in the impact of SMEs’ synergistic transformation of digitization and greenization on risk-taking is not apparent. This paper attempts to answer the above questions, extend the application scope of the Haken model, and provide empirical evidence on the impact of SMEs’ synergistic transformation of digitization and greenization on risk-taking.

(3) **The paper claims that reducing cash holdings and R&D investment enhances risk-taking, but this assumption may not hold universally. Additional robustness checks or alternative measures of risk-taking could strengthen the argument. Kindly offer alternative explanations for why cash holdings and R&D investment reductions lead to increased risk-taking.**

**The response of author:**

Thank you to the reviewers for your comments on the robustness test. In order to enable readers to better understand the main conclusion of this article, the author has reorganized the 5.5 robustness test section. The robustness test of this paper is carried out from two perspectives. 5.5.1 presents the robustness test results after changing the control variables. 5.5.2 presents the results of changing the measurement methods of the dependent variable and the independent variable. The research results are respectively explained in these two parts to better enable readers to understand the robustness test of this study. The main changes are as follows (743-778):

**Changing control variables**

In this paper, control variables were changed to test the robustness of the main study conclusions (as shown in Table 7). Section A of Table 7 shows the results without adding control variables. Section B shows the results of controlling for only financial indicators (including ROE, DEBT, INTEN, and TAT). Section C lists the results of controlling for only the corporate governance indicators (including AGE, BSIZE, SHARE, and DUAL). Column (1) lists the impact of SDG on RISK, and the coefficients are all significantly positive, which verifies that the synergistic transformation of digitization and greenization significantly promotes enterprise risk-taking. Columns (2) and (3) present the mediating effect of CASH. Columns (4) and (5) show the mediating effect of RD. These results validate SMEs’ synergistic transformation of digitization and greenization, which promotes enterprise risk-taking by reducing cash holdings and R&D investments. This paper’s main conclusions have not changed according to the above results.

**Table 7 Change control variable**

| Section A: No control variables | | | | | |
| --- | --- | --- | --- | --- | --- |
|  | (1)  RISK | (2)  CASH | (3)  RISK | (4)  RD | (5)  RISK |
| SDG | .0050**  (2.1891) | -.1070***  (-6.7957) | .0031  (1.3118) | -.0808**  (-2.5455) | .0043*  (1.7504) |
| CASH |  |  | -.0195***  (-10.1366) |  |  |
| RD |  |  |  |  | -.0027***  (-2.6528) |
| constant term | .0087  (.1261) | 23.3877***  (48.4358) | .4657***  (5.4699) | 17.9385***  (17.2254) | .0864  (1.0439) |
| Observations | 7410 | 6995 | 6995 | 6741 | 6741 |
| R^2^ | .0313 | .2783 | .0484 | .1755 | .0326 |
| Section B: Only financial indicators controlled | | | | | |
|  | (1)  RISK | (2)  CASH | (3)  RISK | (4)  RD | (5)  RISK |
| SDG | .0070***  (3.1561) | -.1086***  (-6.9148) | .0054**  (2.3505) | -.0776**  (-2.4419) | .006**  (2.5261) |
| CASH |  |  | -.0167***  (-9.0281) |  |  |
| RD |  |  |  |  | -.0035***  (-3.5949) |
| ROE | -.0044***  (-11.2519) | .0053**  (1.9912) | -.0043***  (-11.0571) | .0027  (.5068) | -.0042***  (-10.6534) |
| DEBT | .1361***  (16.8857) | -.2163***  (-3.7284) | .1516***  (18.053) | .4155***  (3.3596) | .1787***  (19.4606) |
| INTEN | .0008**  (2.4459) | .0001  (.0641) | .0008**  (2.3086) | -.0066  (-1.4776) | .0006*  (1.8883) |
| TAT | .0178***  (3.7595) | -.1866***  (-5.4491) | .0099**  (1.9987) | .0423  (.6088) | .0129**  (2.5055) |
| constant term | -.1425**  (-2.1264) | 23.6483***  (48.7796) | .2461***  (2.9765) | 17.6384***  (16.8667) | -.0433  (-.5448) |
| Observations | 7410 | 6995 | 6995 | 6741 | 6741 |
| R^2^ | .0977 | .2845 | .1211 | .1774 | .1169 |
| Section C: Only governance indicators controlled | | | | | |
|  | (1)  RISK | (2)  CASH | (3)  RISK | (4)  RD | (5)  RISK |
| SDG | .0048**  (2.1234) | -.1041***  (-6.7162) | .0031  (1.3027) | -.0749**  (-2.3576) | .0042*  (1.6849) |
| CASH |  |  | -.0192***  (-9.8236) |  |  |
| RD |  |  |  |  | -.0026**  (-2.5313) |
| AGE | -.0019  (-.1751) | -.2411***  (-3.0673) | .0030  (.2494) | -.1527  (-.9393) | .0074  (.5873) |
| BSIZE | -.0103  (-1.3558) | .0384  (.7471) | -.0104  (-1.3312) | .2881***  (2.7313) | -.0099  (-1.208) |
| SHARE | -.0005***  (-2.8091) | .0163***  (12.7633) | -.0003  (-1.3717) | .0069***  (2.6334) | -.0005***  (-2.7028) |
| DUAL | .0086**  (2.2567) | .1095***  (4.2195) | .0111***  (2.8141) | .1068**  (2.0182) | .0077*  (1.8801) |
| constant term | .0740  (.9372) | 23.0367***  (41.8856) | .4971***  (5.2280) | 17.1780***  (14.4420) | .1225  (1.3043) |
| Observations | 7410 | 6995 | 6995 | 6741 | 6741 |
| R^2^ | .0337 | .3033 | .0503 | .1785 | .0349 |

*** p<.01, ** p<.05, * p<.1

**Change the measurement of main variables**

To test the reliability of the conclusions further, this paper changed the measurement of the main variables (as listed in Table 8). The results of changing the calculation of dependent variables are demonstrated in section A of Table 8. Referring to the practice of Shahzad ^[73]^, Yung & Chen ^[74]^, the standard deviation of the three-year adjusted ROE is used to measure enterprise risk-taking (RISK*) without changing the independent variables and control variables. The results of changing the calculation of independent variables are shown in section B of Table 8. This paper uses the data of all listed companies in China from 2012 to 2022 to calculate the SMEs’ synergistic transformation of digitization and greenization (SDG*) without changing the dependent and control variables. Column (1) lists the impact of SMEs’ synergistic transformation of digitization and greenization on enterprise risk-taking, and the coefficients are all significantly positive. Columns (2) and (3) present the mediating effect of CASH. Columns (4) and (5) show the mediating role of RD. These results validate that the main conclusions have not changed.

**Table 8 Change the measurement of main variables**

| Section A: Change the calculation method of dependent variable | | | | | |
| --- | --- | --- | --- | --- | --- |
|  | (1)  RISK* | (2)  CASH | (3)  RISK* | (4)  RD | (5)  RISK* |
| SDG | .0069***  (3.1476) | -.1049***  (-6.7817) | .0053**  (2.3347) | -.0719**  (-2.2637) | .0059**  (2.5239) |
| CASH |  |  | -.0178***  (-9.538) |  |  |
| RD |  |  |  |  | -.0036***  (-3.6989) |
| ROE | -.0044***  (-11.4581) | .0055**  (2.089) | -.0044***  (-11.3473) | .0027  (.505) | -.0043***  (-10.9403) |
| ROE | -.0044***  (-11.4581) | .0055**  (2.089) | -.0044***  (-11.3473) | .0027  (.505) | -.0043***  (-10.9403) |
| AGE | -.0288***  (-2.7884) | -.1928**  (-2.4364) | -.0302***  (-2.6337) | -.2460  (-1.497) | -.0336***  (-2.788) |
| DEBT | .1421***  (17.5227) | -.1772***  (-3.0737) | .1589***  (18.9792) | .4494***  (3.5954) | .1877***  (20.4523) |
| BSIZE | -.0153**  (-2.0896) | .0465  (.9080) | -.0151**  (-2.0256) | .2743***  (2.6011) | -.0135*  (-1.7409) |
| INTEN | .0009***  (2.7136) | .0011  (.4978) | .0008***  (2.6246) | -.0062  (-1.3763) | .0007**  (2.127) |
| TAT | .0211***  (4.4623) | -.1635***  (-4.8488) | .0141***  (2.8734) | .054  (.7764) | .017***  (3.3365) |
| SHARE | -.0005***  (-2.6839) | .0161***  (12.6515) | -.0003  (-1.5355) | .0069***  (2.6533) | -.0005***  (-2.8012) |
| DUAL | .0076**  (2.0743) | .107***  (4.1371) | .0097***  (2.5852) | .1085**  (2.0511) | .0078**  (1.9994) |
| constant term | -.0113  (-.1479) | 23.1001***  (42.074) | .3958***  (4.3727) | 17.1425***  (14.416) | .1063  (1.1971) |
| Observations | 7410 | 6995 | 6995 | 6741 | 6741 |
| R^2^ | .1059 | .3081 | .1335 | .1807 | .1287 |
| Section B: Change the calculation method of independent variable | | | | | |
|  | (1)  RISK | (2)  CASH | (3)  RISK | (4)  RD | (5)  RISK |
| SDG* | .0065***  (3.0175) | -.1026***  (-6.7804) | .005**  (2.2466) | -.0703**  (-2.2654) | .0055**  (2.3772) |
| CASH |  |  | -.0168***  (-8.8977) |  |  |
| RD |  |  |  |  | -.0035***  (-3.5538) |
| ROE | -.0044***  (-11.2637) | .0055**  (2.089) | -.0043***  (-11.066) | .0027  (.5050) | -.0042***  (-10.6902) |
| AGE | -.0276***  (-2.6643) | -.1928**  (-2.4363) | -.0283**  (-2.4384) | -.2460  (-1.4971) | -.0326***  (-2.676) |
| DEBT | .1389***  (17.0943) | -.1772***  (-3.0735) | .1546***  (18.2566) | .4494***  (3.5954) | .1823***  (19.6414) |
| BSIZE | -.0152**  (-2.0648) | .0465  (.9081) | -.0152**  (-2.018) | .2743***  (2.601) | -.0138*  (-1.7612) |
| INTEN | .0008**  (2.3902) | .0011  (.4978) | .0007**  (2.2981) | -.0062  (-1.3763) | .0006*  (1.8335) |
| TAT | .0180***  (3.8028) | -.1635***  (-4.8488) | .0104**  (2.1065) | .0540  (.7764) | .0133***  (2.5816) |
| SHARE | -.0005**  (-2.5434) | .0161***  (12.6512) | -.0003  (-1.4847) | .0069***  (2.6532) | -.0005***  (-2.6827) |
| DUAL | .0088**  (2.383) | .1071***  (4.1374) | .0107***  (2.8036) | .1085**  (2.0512) | .0087**  (2.224) |
| constant term | .0213  (.2983) | 22.7647***  (44.2115) | .3970***  (4.5692) | 16.9143***  (15.0751) | .1304  (1.5375) |
| Observations | 7410 | 6995 | 6995 | 6741 | 6741 |
| R^2^ | .1008 | .3081 | .1240 | .1807 | .1200 |

*** p<.01, ** p<.05, * p<.1

This article holds that SMEs synergistic transformation of digitization and greenization promote risk taking by reducing cash holdings and R&D investment. The reason lies in:

On the one hand, according to the agency theory, having the same goals as shareholders and management helps enterprises concentrate superior resources to implement low-cost and high-efficiency investment activities and reduce the cash holding by management in pursuit of private gains (Amess et al., 2015). On the other hand, according to information asymmetry theory, good information transparency helps alleviate the agency problem between shareholders and creditors and reduces the cash holding by SMEs due to preventive motives. The SMEs synergistic transformation of digitization and greenization can provide the outside world with more abundant and effective information, attract more effective financial support for digital and green investment, reduce the cash holding due to preventive motives, improve the investment efficiency, and thereby increase the risk assumption. Therefore, The SMEs synergistic transformation of digitization and greenization can reduce cash holdings by lowering agency costs, enhance the investment efficiency in digitalization and greenization, and increase their risk taking.

According to synergetics, synergistic transformation of digitization and greenization can enable digital knowledge and green knowledge to achieve the maximum effect through competition and cooperation, promoting knowledge spillover and the creation of new knowledge. Under the background of the digital economy and the green economy, enterprises continuously promote digital transformation and green transformation (Hess, 2016; Wang et al., 2022). The integration of digital knowledge and green knowledge helps enterprises concentrate their superior resources to carry out innovative activities and constantly seek coordination and cooperation in terms of digital and green innovation investment. Concentrated investment in innovation helps to reduce the R&D costs, improve the efficiency of enterprise innovation investment, and thereby enhance the risk taking.

(4) **Provide more transparency on data sources, keyword selection validation, and the paper does not indicate whether the datasets or analysis scripts will be made available for replication.**

**The response of author：**

Thank you for the comments raised by the reviewers. Providing replicable data sources plays a very important role in promoting research in this field. The main data of this paper were derived from the CSMAR database and the annual reports of listed companies of SMEs, and python was used to crawl the annual report information. The dataset used in this paper has been submitted as supporting information in the system, and the new instrument variable are added. Interested readers can download and replicate it.
