# Supplementary material for: Can SMEs synergistic transformation of digitization and greenization promote enterprise risk-taking?
Source: PLoS One. 2025 Dec 11;20(12):e0337815. doi: 10.1371/journal.pone.0337815 (PMC12698014; doi:10.1371/journal.pone.0337815)
Supplement: Attachment — Submitted filename: Response_to_Reviewers_auresp_2.docx [file pone.0337815.s002.docx]

Dear academic editor and reviewers:

Thank you for taking the precious time to review my manuscript. These valuable comments are very helpful for improving the quality of the paper. Over the past three weeks, I have revised the format of the paper and standardized the writing style words by words. These revisions are of great help to the quality of the paper. The responses to the academic editor are as follows:

**To academic editor**

**Comments : The paper needs a recheck of English grammar. Also, please improve paragraph transitions, and ensure consistent citation formatting throughout the manuscript.**

**The response of author：**
Thank you for the comments of the academic editor. I revised the entire paper in terms of grammar, the connection between sentences and paragraphs, and the format of citation, to make all of these more in line with English usage. All revised sentence are marked in yellow in *Revised Manuscript with Track Changes*. Thanks a lot.
